# Human Leptospirosis on Reunion Island, Indian Ocean: Are Rodents the (Only) Ones to Blame?

**DOI:** 10.1371/journal.pntd.0004733

**Published:** 2016-06-13

**Authors:** Vanina Guernier, Erwan Lagadec, Colette Cordonin, Gildas Le Minter, Yann Gomard, Frédéric Pagès, Marie-Christine Jaffar-Bandjee, Alain Michault, Pablo Tortosa, Koussay Dellagi

**Affiliations:** 1 Centre de Recherche et de Veille sur les maladies émergentes dans l’Océan Indien (CRVOI), Plateforme de Recherche CYROI, Sainte Clotilde, Reunion Island, France; 2 Université de La Réunion, UMR PIMIT "Processus Infectieux en Milieu Insulaire Tropical", INSERM U1187, CNRS 9192, IRD 249. Plateforme de Recherche CYROI, Sainte Clotilde, Reunion Island, France; 3 Regional Office (Cire) of the French Institute for Public Health Surveillance (Institut de veille sanitaire), Reunion Island, France; 4 Laboratory of Virology, Centre Hospitalier Universitaire/CHD, Saint-Denis, Reunion Island, France; 5 Laboratory of Biology, Centre Hospitalier Universitaire/GHSR, Saint-Pierre, Reunion Island, France; University of California San Diego School of Medicine, UNITED STATES

## Abstract

**Background:**

Although leptospirosis is a zoonosis of major concern on tropical islands, the molecular epidemiology of the disease aiming at linking human cases to specific animal reservoirs has been rarely explored within these peculiar ecosystems.

**Methodology/Principal Findings:**

Five species of wild small mammals (n = 995) as well as domestic animals (n = 101) were screened for *Leptospira* infection on Reunion Island; positive samples were subsequently genotyped and compared to *Leptospira* from clinical cases diagnosed in 2012–2013 (n = 66), using MLST analysis. We identified two pathogenic species in human cases, namely *Leptospira interrogans* and *Leptospira borgpetersenii*. *Leptospira interrogans* was by far dominant both in clinical samples (96.6%) and in infected animal samples (95.8%), with *Rattus spp* and dogs being its exclusive carriers. The genetic diversity within *L*. *interrogans* was apparently limited to two sequence types (STs): ST02, identified among most clinical samples and in all rats with complete MLST, and ST34, identified in six humans, but not in rats. Noteworthy, *L*. *interrogans* detected in two stray dogs partially matched with ST02 and ST34. *Leptospira borgpetersenii* was identified in two clinical samples only (3.4%), as well as in cows and mice; four haplotypes were identified, of which two seemingly identical in clinical and animal samples. *Leptospira borgpetersenii* haplotypes detected in human cases were clearly distinct from the lineage detected so far in the endemic bat species *Mormopterus francoismoutoui*, thus excluding a role for this volant mammal in the local human epidemiology of the disease.

**Conclusions/Significance:**

Our data confirm rats as a major reservoir of *Leptospira* on Reunion Island, but also pinpoint a possible role of dogs, cows and mice in the local epidemiology of human leptospirosis. This study shows that a comprehensive molecular characterization of pathogenic *Leptospira* in both clinical and animal samples helps to gaining insight into leptospirosis epidemiology within a specific environmental setting.

## Author Summary

Leptospirosis is a zoonosis caused by infection with pathogenic *Leptospira* species. A broad range of animals, including rodents, pets and livestock, are maintenance hosts for leptospires. However, assessing the relative importance of each host in the contamination of the environment and, *in fine*, in the infection of humans, has rarely been performed. In this study, we surveyed various wild and domestic animal species and their *Leptospira* carriage in Reunion Island, where human leptospirosis is endemic. We determined and compared the *Leptospira* genetic diversity at the species and infra-species levels in laboratory-confirmed human cases and in infected animals. The two *Leptospira* species infecting humans, *Leptospira interrogans* and *Leptospira borgpetersenii*, could be traced back to different animal species: rats and dogs for the former species, cows and mice for the latter. The *Leptospira* infecting the single bat species endemic to the island was not found to be involved in human leptospirosis. Aside from rats, which were expected to play a role in the local epidemiology of the disease, the putative role of dogs, cattle and mice in human epidemiology on Reunion Island, pinpointed by our data, deserves a specific investigation. These results have strong implications in terms of local control actions aimed at reducing the burden of human leptospirosis.

## Introduction

Leptospirosis is a bacterial systemic infection, occasionally fatal, caused by the pathogenic spirochetes of the genus *Leptospira*. Though claimed as the most widespread zoonosis in the world [1,2], the disease is considered as emerging in many parts of the world. Leptospirosis is most prevalent in tropical and subtropical countries [3,4], presumably because the survival of the bacterium outside the host requires humid and warm conditions that are typical of tropical areas [5]. Rodents are recognized as the main reservoir of pathogenic *Leptospira* although several animal species are also capable of sustaining biofilm colonization of the renal tubules and shedding the bacteria in their urine [6]. Humans usually get infected through indirect exposure with water or soil contaminated with urine, but direct transmission has also been suggested as important for some species [7].

Traditionally, *Leptospira* have been classified serologically into 25 serogroups and over 300 serovars using Microscopic Agglutination Test (MAT) and Cross agglutination absorption test (CAAT) analysis, respectively [1,8]. More recently, a genetic classification based on DNA-DNA hybridization complemented by molecular methods and experimental studies have confirmed the existence of at least 22 *Leptospira* species [9]. The congruence between serological and molecular classifications is poor [1]: one serogroup can be linked to several *Leptospira* species while serovars can vary within a given clone or lineage, an observation considered as probably resulting from horizontal gene transfer [10]. Among the various molecular tools currently used to genotype *Leptospira*, multilocus sequence typing (MLST) has emerged as a method of choice as it provides data produced at a local scale that can be further compared to genotypes obtained all over the world and made freely accessible to the scientific community through public databases.

Leptospirosis is endemic in several islands of the southwestern Indian Ocean (SWIO) including the two French overseas departments of Mayotte [11,12] and Reunion Island [13,14] together with the island state of Seychelles [14–16], the latter recording the highest human incidence reported worldwide [17]. On Madagascar, serological evidence of exposure to leptospirosis has been reported in the human community of Moramanga [18], while to our knowledge, a single case of acute leptospirosis infection has been PCR-confirmed on a traveller returning from Madagascar [19]. Only serological evidence of human exposure to pathogenic *Leptospira* has been reported on the three neighboring islands of Union of the Comoros [20], while no or only scarce data is available for Mauritius [21] and Rodrigues Islands. On Mayotte, improved diagnostic procedures allowed to significantly increase the number of confirmed cases, with about 100 new human cases reported each year since 2009 [12,22]. The MLST analysis of *Leptospira* isolated from human incident cases on the island revealed a large bacterial diversity of clinical isolates, represented by at least 17 different “sequence types” (STs) and four distinct *Leptospira* species, namely *Leptospira interrogans*, *Leptospira borgpetersenii*, *Leptospira kirschneri*, and the newly described *Leptospira mayottensis* [12,23].

Comparatively, information is more abundant on pathogenic *Leptospira* infecting wild animals from the SWIO. On Mayotte, sequencing of a portion of the 16S rRNA (*rrs*) gene of leptospires infecting twenty black rats *Rattus rattus* has identified the same four *Leptospira* species previously reported in clinical cases from the same island, with a strict identity between the sequences of *Leptospira* infecting *R*. *rattus* and humans [11], designating this rodent species as the probable major reservoir and transmission source of *Leptospira* to humans on Mayotte [11]. On Comoros and Madagascar, sequencing of the nearly complete 16S rRNA gene of *Leptospira* infecting kidneys from six bat species (n = 7) has shown the carriage of *L*. *interrogans* (Comoros), *L*. *borgpetersenii* (Comoros and Madagascar) and other thus far unknown *Leptospira* species [24]. On Madagascar, partial sequencing of *rrs* locus has identified a single *L*. *interrogans* haplotype among 70 samples from introduced small mammals (rats, shrews and mice) [25]. Only one full five gene-based MLST analysis has been reported so far from the SWIO region, i.e. on Madagascar, where authors identified *L*. *borgpetersenii*, *L*. *mayottensis* and *L*. *kirschneri* from endemic small mammals and bats [26]. This study revealed distinct clustering associated with host type, with no overlap between *Leptospira* species infecting endemic small mammals versus those infecting introduced ones, a feature which certainly deserves further investigation.

On Reunion Island, human leptospirosis was first reported in 1953 but the first outbreak most likely occurred in 1868 [27]. Since 1953, several studies have been conducted to assess the burden of leptospirosis on the island. From 2008 to 2012, the mean annual incidence was estimated at 8.2 cases per 100,000 inhabitants with a fatality rate around 4%, and Icterohaemorrhagiae identified as the major serogroup in severe forms [13]. Lower prevalences of serogroups Canicola, Grippotyphosa and Australis have also been reported [28]. Pagès and colleagues [13] have highlighted the following population groups as being at highest risk: farmers and green space workers, people under 20 years old and participating in aquatic activities, people between 20 and 30 years old that fish, and people between 50 and 60 year old gardening at home. The seroprevalence of infection and/or *Leptospira* carriage in potential reservoirs, have been explored in wild small mammals (rodents, shrews, tenrecs and bats) and domestic animals (dogs, cats, cattle, goats, swine, rusa deers and horses) [14,29–32]. Since then, the black rat, *Rattus rattus*, abundant in most regions and biotopes of the island, has been considered to be the primary reservoir and transmitter of *Leptospira spp*., a conclusion mainly based on the observation that Icterohaemorrhagiae is by far the main serogroup found in rats and clinical cases [29], though the same serogroup has also been identified in dogs, pigs, rusa deer and tenrecs [29]. Recently, the first molecular study from Reunion Island identified that patients with acute leptospirosis (n = 42) were all infected by *L*. *interrogans* [33]. Although the typing method, High Resolution Melting (HRM) analysis of two VNTR sequences, allows a rapid diagnosis on clinical samples together with the characterization at the serovar and species levels, it is likely not resolutive enough as to trace the source(s) leading to human infection and illness.

The aim of our study was to determine the wild and/or domestic animal species that not only serve as reservoir hosts for pathogenic *Leptospira* species, but also are primary sources for human infection on Reunion Island, an area considered as endemic for human leptospirosis.

## Materials and Methods

### Human samples

Sixty-six patients whose blood samples tested positive for *Leptospira* (see details hereafter) were included in the study. Sera were collected for diagnostic purpose from patients originated from all four sanitary regions recognized on the island and admitted for acute febrile syndrome in 2012–2013 to either of the two main University hospitals of La Reunion (northern and southern University hospitals, hereafter referred to as NUH and SUH respectively). All patients included in the study were considered as autochthonous leptospirosis cases as none of them reported travel history during the month preceding the onset of symptoms.

### Animal samples

**Fig 1:**
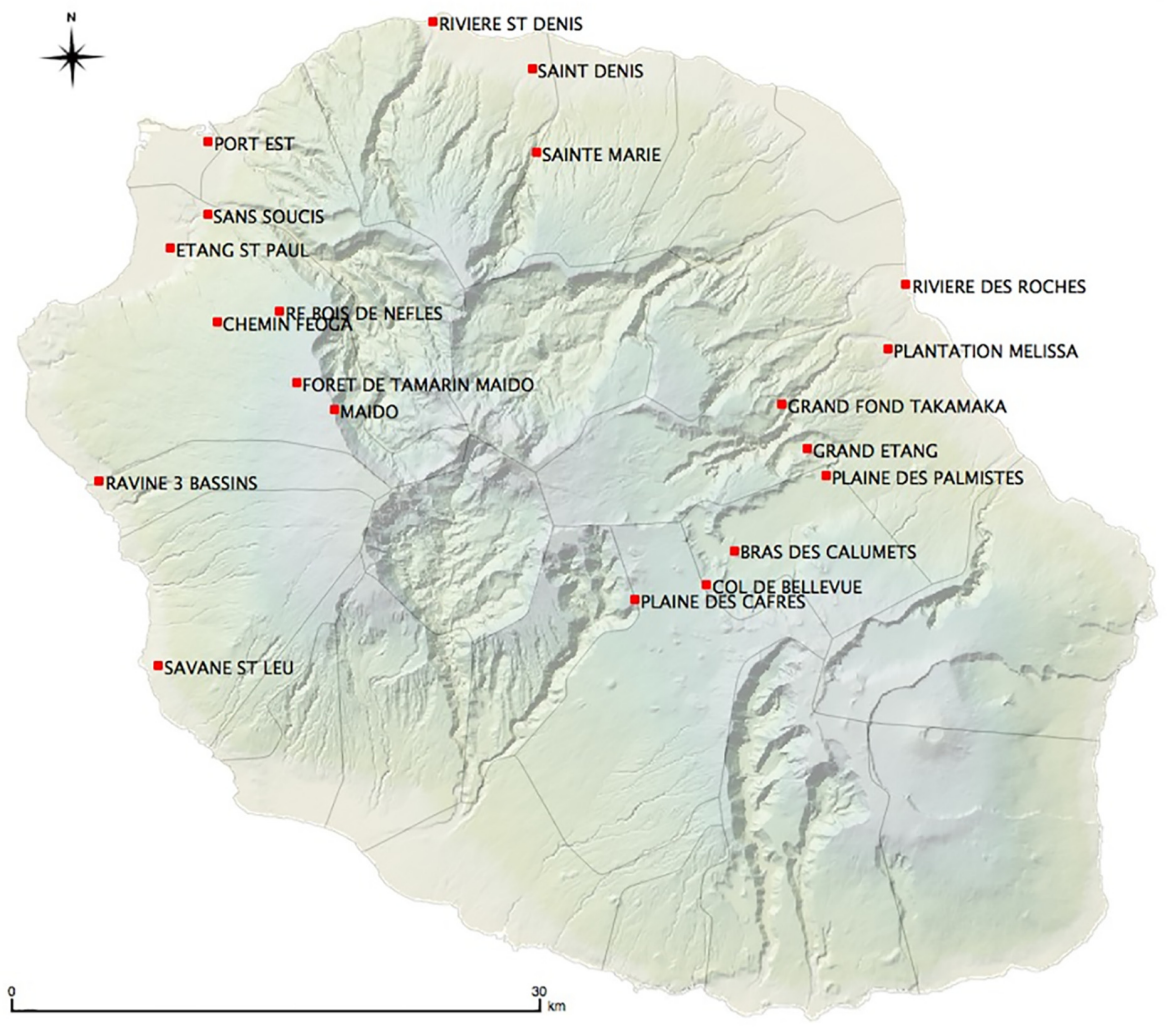
Sampling sites along the two altitudinal transects on western and eastern coasts, together with additional sampling sites in the north and west coast of Reunion Island.

We trapped 799 rodents (562 *Rattus rattus*, 170 *Rattus norvegicus*, 67 *Mus musculus domesticus*), 171 shrews (*Suncus murinus*) and 25 tenrecs (*Tenrec ecaudatus*), those species representing all terrestrial small mammal diversity occurring on Reunion Island. Trapping occurred in 2012–2014 during dry and rainy seasons on twenty sampling sites, fifteen of them selected along two altitudinal transects on wet windward and dry leeward coasts (Fig 1). Details on trapping of wild animals and study sites have been published elsewhere (see [34]). Kidneys from cows (n = 33) and pigs (n = 22) were directly collected at the unique slaughterhouse of the island, which was not able to provide information regarding the geographic origin of the samples. Kidneys from stray dogs (n = 45) were collected just after the animals were euthanized at the community pound of Saint André (eastern coast). The only cat sample included in the study was urine collected by a veterinarian.

### Ethics statement

The ethical terms of the research protocol were approved by the CYROI institutional ethical committee (Comité d’Ethique du CYROI n°114, IACUC certified by Ministry of Higher Education and Research) and by the Ministry of higher education and research under accreditation 03387.01 (LeptOI). All animal procedures carried out in our study were performed in accordance with the European Union legislation for the protection of animals used for scientific purposes (Directive 2010/63/EU). The stray dogs were euthanized by lethal injection administered by a veterinarian in the frame of population control measures implemented by the local authorities. Residual sera from anonymized clinical samples collected for diagnostic purposes and laboratory-confirmed as leptospirosis cases were obtained from the clinical laboratories of NUH and SUH.

### PCR detection for diagnosis

Genomic DNA was extracted from human sera at hospital laboratories using NucliSENS easyMAG system (BioMérieux, Marcy l’Etoile, France) (NUH) or Dneasy Blood and Tissue Kit (Qiagen, Courtaboeuf, France) (SUH) according to the manufacturer’s instructions. The presence of *Leptospira* was assessed with a probe-specific real-time PCR targeting either (i) the 23S rRNA gene [35] in NUH or (ii) LA0322 locus (LFB1 primers) [36] in SUH.

Extraction of total nucleic acids from animal samples was performed from a pool of kidney, lung and spleen tissues (for wild animals) or from kidneys only (for domestic animals) using the Biorobot EZ1 and EZ1 Virus Mini Kit version 2.0 (QIAGEN, Les Ulis, France). A reverse transcription step was then performed with GoScript reverse transcriptase (Promega, Charbonnières-les-Bains, France); generated cDNA was used as template for *Leptospira* detection through a previously described probe-specific real-time PCR targeting the *rrs* (16S rRNA) locus [24,37]. Animal samples leading to a PCR amplification at Ct < 42 were considered as positive.

### *Leptospira* isolation

Twenty-four animal samples were randomly selected in order to attempt *Leptospira* isolation from kidney cultures, i.e. 15 *R*. *rattus*, two *R*. *norvegicus*, two *M*. *musculus*, three *S*. *murinus* and two *T*. *ecaudatus*. A small piece of the freshly sampled kidney was crushed under sterile conditions and used to inoculate three distinct culture media: (i) Ellinghausen-McCullough-Johnson-Harris (EMJH) liquid medium (Difco, Detroit, MI, USA) supplemented with Albumin Fatty Acid Supplement (AFAS; Royal Tropical Institute, Amsterdam, Netherlands) [38,39]; (ii) EMJH liquid medium supplemented with AFAS, rabbit serum and foetal calf serum (1% each); and (iii) semisolid Fletcher medium (Difco, Detroit, MI, USA) supplemented with rabbit serum (8%). All media were supplemented with 5-fluorouracil (5-FU) at a final concentration of 200 μg.mL^-1^. Cultures were incubated at 28°C, visually checked for the presence of *Leptospira* using a dark field microscope once a week for four months, and positive cultures were further sub-cultured in fresh EMJH liquid medium deprived of 5-FU. DNA was extracted from 1 mL of each positive culture using the EZ1 Biorobot with Qiagen EZ1 DNA Tissue kits (Qiagen, Les Ulis, France).

### *Leptospira* genotyping

Three major MLST schemes exist for *Leptospira* spp. typing worldwide, all supported by the website database http://pubmlst.org/leptospira/. In our study, scheme #3 [40] was preferred to the other two [10,41] as several molecular data from the SWIO have been made freely available using this same scheme, hence allowing comparison of local STs with those from SWIO islands [12,26]. MLST was attempted for *adk*, *icdA*, *lipL32*, *rrs2*, *secY*, and *lipL41* loci as previously described [40] and recently optimized [26]. All positive domestic animal samples were first submitted to *secY* sequencing, revealing a nearly clonal population among infected rats (see Results). Hence, a randomly selected subgroup of positive wild animal samples was selected from eleven sites (n = 17; Table 1) and submitted to full MLST genotyping.

**Table 1:** Leptospirosis testing results by host species. *Leptospira* prevalence after real-time PCR diagnosis; species identification, either by 16S rRNA or *secY* sequencing and rate of success; and *secY* or MLST amplification and sequencing success.

	*Leptospira* prevalence (%)	Identification success (%)	Infecting Leptospira	*secY* amplification ^a^	6-loci (or 5 ^b^) MLST
Cattle	14/33 (42.4)	8/14 (57.1)	*Lb* (n = 8)	5	0/5 (0)
Dog	7/45 (15.6)	2/7 (28.6)	*Li* (n = 2)	2	0/2 (0)
Pig	0/22 (0)	-	-	-	-
Cat	1/1 (100)	0/1 (0)	-	-	-
Domestic animals	22/101 (21.8)	10/22 (45.4)	*Lb* (n = 8), *Li* (n = 2)	7	0/7 (0)
*RR*	214/562 (38.5)	201/214 (93.9)	*Li* (n = 201)	164	4/11 (9)
*RN*	52/170 (30.6)	45/52 (86.5)	*Li* (n = 45)	27	1/6 (2)
*SM*	6/171 (3.5)	3/6 (50)	*Li* (n = 3)	-	-
*MM*	6/67 (9.0)	3/6 (50)	*Lb* (n = 1), *Lk* (n = 2)	--	--
*TE*	0/25 (0)	-	-	-	-
Wild animals	278/995 (27.9)	252/278 (90.6)	*Lb* (n = 1), *Li* (n = 249), *Lk* (n = 2)	191	5/17 (11)
Human	-	59/66 (89.4)	*Li* (n = 57), *Lb* (n = 2)	48-	44/48 (44)-

RR: Rattus rattus; RN: Rattus norvegicus; SM: Suncus murinus; MM: Mus musculus; TE: Tenrec ecaudatus. Li: Leptospira interrogans; Lb: Leptospira borgpetersenii; Lk: Leptospira kirschneri.

^a^ This excludes partial *secY* amplification (< 501 bp) of four clinical samples and one cow sample;

^b^ Amplification and sequencing success for the 5-loci MLST scheme excluding the locus *icdA*.

For species identification of other positive wild animal samples, including two culture isolates, the *secY* gene was amplified with the primers secY F/R [40] or alternatively G1/G2 [42]. When *secY* PCR amplification failed, a 16S rRNA gene sequencing was alternatively used with previously published primers, i.e. LA/LB [43] or LA/R2 [44].

**Fig 2:**
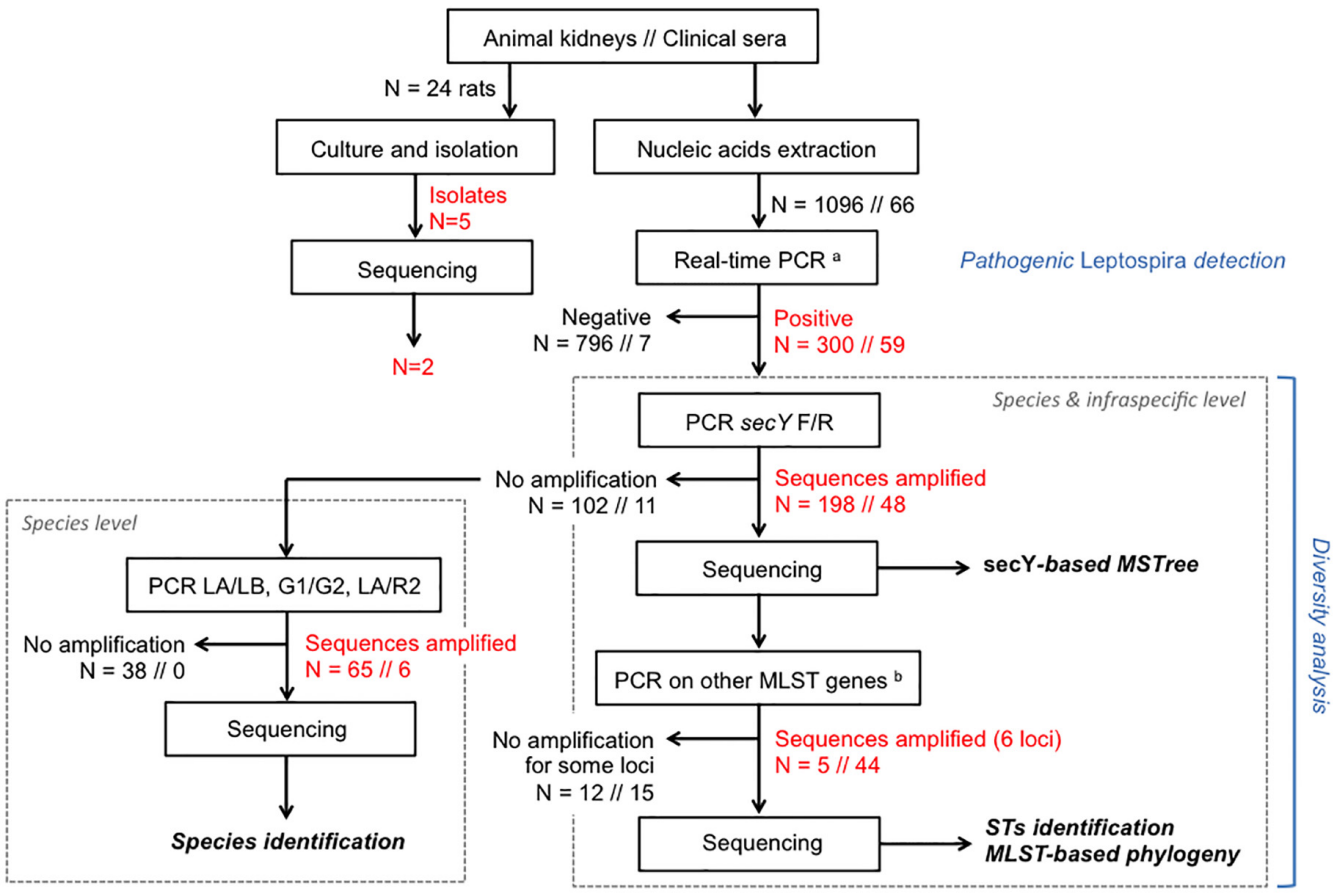
Flow chart of the molecular methods performed to detect and identify *Leptospira* species and haplotypes infecting samples from Reunion Island. At each step, the number (N) of tested animal samples // clinical samples is indicated. ^a^ The real time PCR diagnosis was attempted on either DNA extracted from clinical samples, or cDNA obtained after a reverse transcription step for animal samples. ^b^ Six-loci MLST was attempted only for a selected subgroup of the 198 animal samples successfully amplified on *secY* locus using MLST scheme #3, i.e. eight domestic animals and 17 randomly selected wild small mammals (all *Rattus* spp).

Obtained amplicons were directly sent for direct Sanger sequencing on both strands (Genoscreen, Lille, France), or alternatively cloned into the pGEM-T easy vector (Promega, Madison, WI, USA) when the quantity was insufficient for direct sequencing. Obtained clones (five per sample) were subsequently sequenced using M13 universal primers for Sanger sequencing. The overall analysis strategy is presented in Fig 2.

### Phylogenetic analysis

Consensus sequence for each sample and multiple alignments between sequences were obtained using Geneious Pro version 5.4 [45]. In order to provide a comparison with *Leptospira* strains occurring worldwide, our dataset included sequences from two human clinical isolates from Mayotte [12] and 118 human and animal isolates from all over the world [2]. Sequence alignments were constructed separately for all six considered loci. A five-loci concatenate was generated using SEQMATRIX v1.7.8 [46], excluding *icdA* locus that was unavailable from the Mayotte clinical dataset and for some of our samples. Bayesian analyses were performed to infer phylogenetic relationships between *Leptospira* species. The best-fitting model and associated parameters were selected using jModelTest [47] and phylogenies were constructed by Bayesian inference. We performed two independent runs of Metropolis-coupled Markov chain Monte Carlo (MCMCMC) analyses in MrBayes v3.2.1 [48] of all loci independently and of concatenated sequences. Each run included 20,000,000 generations, and trees were sampled every 100 generations. The initial 20,000 trees were discarded as a conservative "burn-in" and the harmonic mean of the likelihood was calculated by combining the two independent runs. The 50% majority-rule consensus tree was then computed from the sampled trees under the best model. Neighbor-joining trees were constructed using Seaview v4.3 (Kimura’s 2-parameter distances, 500 replicates). Trees were visualized in FigTree v1.3.1 (http://tree.bio.ecd.ac.uk/). GenBank accession numbers of the sequences produced in the frame of the present study are provided as additional table 1 (S1 Table).

Unique allele identifiers for all six loci were assigned, and corresponding allelic profiles (or sequence types STs) were defined using the established *Leptospira* MLST website (http://pubmlst.org), focusing on MLST scheme #3. *Leptospira*-positive samples with incomplete *Leptospira* MLST cannot be assigned to a ST. In order to determine the DNA relatedness among *Leptospira* carried in the human or animal specimens, we drew a minimum-spanning tree (MST) based on a 501 bp *secY* gene fragment, using the goeBURST Full MST algorithm (goeburst.phyloviz.net/) [49].

## Results

### *Leptospira* diversity within clinical samples

**Fig 3:**
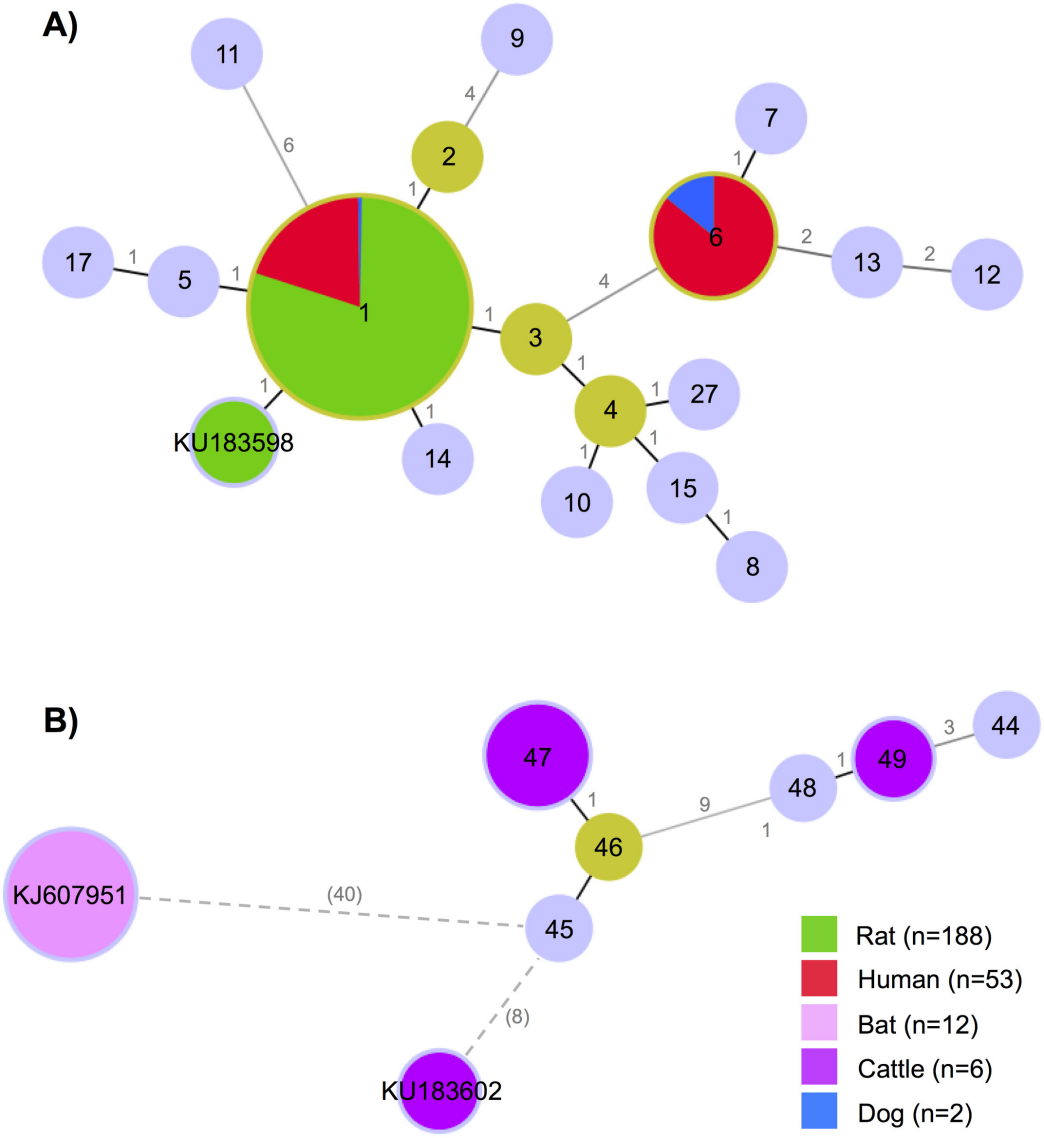
Minimum spanning trees of *Leptospira* based on *secY* gene (501 bp sequence), including our sequences from Reunion Island and twelve bat sequences previously published [46]. A) Minimum spanning tree of *Leptospira interrogans* (n = 257) and B) *Leptospira borgpetersenii* (n = 22). We used the goeBURST Full MST algorithm. The alleles are identified by a number (*secY-*1 to *secY-*54, see http://www.pubmlst.org/leptospira/) or an identifier; the circle size reflects their abundance in the data set. Group founders are in light green and common nodes are in light blue, except when overlapping with alleles from our sample, with specific colours referring to hosts (see legend). Links between the elements uses a grayscale, with lighter gray links showing more differences; the number of differences is indicated on the links. For two incomplete *secY* sequences (< 501 bp), the numbers of differences, shown in parentheses, were calculated on the overlapping nucleotides only, i.e. 443 for KU183602, and 491 for KJ607951.

**Fig 4:**
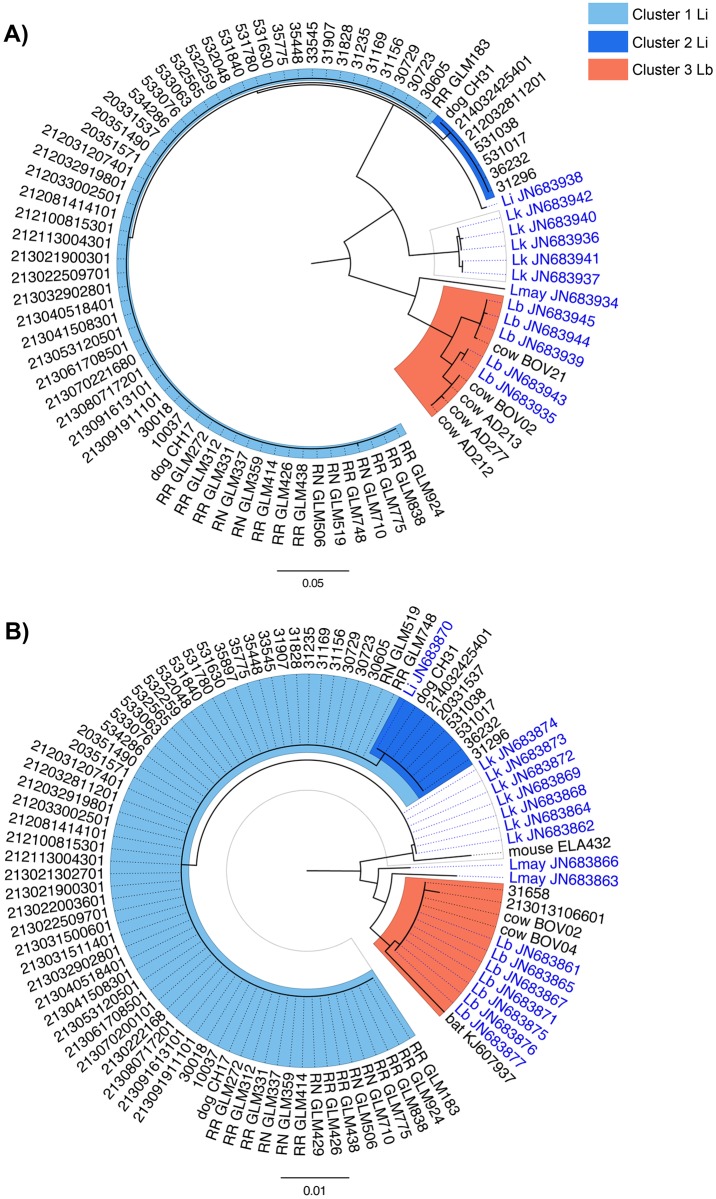
Neighbour-joining phylogenetic trees (Kimura’s 2-parameter distances, 500 replicates) inferred from (A) *secY* (501 bp sequence) and (B) *rrs2* (450 bp sequence) genes. Clinical and animal samples from Reunion Island are written in black, using GenBank accession numbers (for one published bat sequence) or identifiers accompanied by the host name in the case of animal samples (RR: *Rattus rattus*; RN: *Rattus norvegicus)*. Corresponding GenBank accession are reported on S1 Table (supporting information). Clinical samples from Mayotte are written in blue, using GenBank accession numbers and corresponding *Leptospira* species. Li: *Leptospira interrogans*; Lb: *Leptospira borgpetersenii;* Lk: *Leptospira kirschneri*; Lmay: *Leptospira mayottensis*.

For 44 of the 66 clinical samples, we successfully amplified all six MLST loci (Table 1). For twelve clinical samples, only one to five MLST loci could be successfully amplified, even when using degenerated primers [26] (see S2 Table for details). For the last seven clinical samples, we failed to obtain successful PCR amplification at any of the six MLST loci. When these seven samples were further re-tested through an alternative real time PCR [37], only two samples tested positive at Ct values exceeding 42, whereas five sera provided negative amplification, suggesting that these were either false positive samples or positive samples that degraded during transport or conservation. Those seven human negative samples were discarded from the analysis and were no longer considered (Fig 2). The twelve partially sequenced samples allowed only identification of the infecting *Leptospira* at the species level; samples for which *secY* PCR product could be amplified were included in the MST analyses. *In fine*, of the 59 clinical samples successfully amplified at one or more locus, 57 were assigned to *L*. *interrogans* and two to *L*. *borgpetersenii* (Table 1). The *secY*-based MST (Fig 3), the neighbor-joining trees based on *secY* and *rrs2* (Fig 4), and the MLST analysis all showed two *L*. *interrogans* clusters. As the two *L*. *borgpetersenii-*positive clinical samples allowed PCR amplification on two or three loci only (*i*.*e*. *lipL32* and *rrs2* for both samples, *lipL41* for one sample only) but not on *secY* locus, they do not appear in the *secY*-based MST or the MLST-deduced phylogeny.

### *Leptospira* detection and identification in animal samples

Out of 995 wild terrestrial small mammals trapped on Reunion Island, 278 animals (27.9%) tested positive for *Leptospira*. Out of 24 wild terrestrial small mammals for which culture was attempted, five were tested positive for *Leptospira*, among which two were successfully cultured (both *R*. *rattus*). Positive samples amplified and sequenced on *secY* locus provided sequences for 191/278 (68.7%) individuals, all of them being *Rattus* spp. and including the two successfully cultured samples. The remaining 87 positive samples were further tested for each of the other five MLST loci, as well as for small portions of the 16S region. This effort allowed identifying the *Leptospira* species infecting 61 additional samples from wild animals. However, because of the lack of either *secY* sequence or full MLST genotyping, these samples were excluded from the diversity analyses at the infra-specific level. Altogether, 249 out of the 252 *Leptospira* sequences that were obtained from wild mammal samples were identified as *L*. *interrogans* (98.8%). The three remaining sequences, all from mouse tissue samples, were identified either as *L*. *borgpetersenii* (n = 1) or *L*. *kirschneri* (n = 2); the *L*. *borgpetersenii* sample allowed *lipL32* amplification while *rrs2* amplification was obtained from one of the two *L*. *kirschneri* samples. The last *L*. *kirschneri* sample did not lead to any PCR amplification at any of the six MLST loci but was identified at the species level using LA/LB primers. Noteworthy, all *T*. *ecaudatus* tested PCR negative. These results are summarized in Table 1. Interestingly, when aligning the 191 available *secY* sequences, all from rat tissue samples, it appeared that a single *L*. *interrogans secY* allele (*secY*-1) was present in all rats but two. The two exceptions (both *R*. *norvegicus*) had different single substitutions at the *secY* sequence. The amino acid translation showed an internal stop codon in one of the two sequences, suggesting a sequencing error, and this sequence was later excluded. Thus, just one undescribed allele remained (KU183598; see Fig 3).

The 101 domestic animals screened in our study provided the following positivity rates for *Leptospira*: 42.4% in cows, 15.6% in dogs and 0% in pigs (Table 1). The single sample from a cat was urine tested positive for *Leptospira*. Sequences on different loci were obtained for ten animals (S1 Table). *Leptospira* infecting eight cows were identified as *L*. *borgpetersenii*, whereas *Leptospira* infecting two dogs were identified as *L*. *interrogans*, each dog showing a different *secY* allele, i.e. *secY*-6 (also identified in six clinical samples) and *secY*-1 (highly dominant in both clinical and rat samples) (Fig 3).

### Leptospira borgpetersenii diversity

*Leptospira* samples that were identified as *L*. *borgpetersenii* (see Table 1) did not allow PCR amplification at most MLST loci, even when using recently described degenerated primers [26]. For cows (n = 8), we could amplify either *adk* (n = 4), *lipL32* (n = 1), *rrs2* (n = 3), or *secY* locus (n = 6), depending on samples (see S1 Table), whereas only *lipL32* locus was successfully amplified for the single *L*. *borgpetersenii* positive mouse. As for clinical samples (n = 2), we could amplify either *lipL32* (n = 2), *lipL41* (n = 1) or *rrs2* (n = 2). Thus, the only locus that allowed a comparison between human and animal samples was a 450 bp *lipL32* PCR fragment amplified from two clinical samples, one mouse and one cow (S1 Table). Alignments showed 100% nucleotide identity between *L*. *borgpetersenii* infecting the first clinical sample and the only mouse positive for *L*. *borgpetersenii*, and between *L*. *borgpetersenii* infecting the second clinical sample and one cow sample (based on a partial 434 bp amplification for the cow).

The *secY* locus amplification and sequencing provided sequences for six cow samples, revealing alleles *secY*-47 (n = 4), *secY*-49 (n = 1), and a third not yet described allele in one sample (KU183602, see Fig 3). The allele *secY*-47 is closely related to *secY*-48 identified in clinical samples from Mayotte, whereas *secY*-49 has been identified in *Leptospira-*positive Tanzanian rodent samples and in a clinical sample from China (comparison with sequences from [2]). The third undescribed *secY* allele was identified in the cow sample showing a *lipL32* sequence common to those from one clinical sample. Noteworthy, twelve *secY Leptospira* sequences from the insectivorous bat *Mormopterus francoismoutoui* endemic to Reunion Island (GenBank accession numbers KJ607946 to KJ607957) [50] were not related to any of these alleles (Fig 3).

### Genetic diversity using MLST analysis

For 44 clinical samples and eleven rats, five to six loci of the MLST scheme [40] were successfully amplified, sequenced and concatenated for subsequent analyses (Fig 2, Table 1 and S1 Table). The 44 clinical samples which allowed successful amplification at all six MLST loci were identified as *L*. *interrogans* and fell into two clusters corresponding to previously described sequence types (STs): one predominant, ST02 (38/44 = 86.4%) and one minor, ST34 (6/44 = 13.6%). Including samples with successful amplification at 5 MLST loci only, we identified two clonal complexes, CC02 and CC34, including human and rats for the former, and human and dogs for the later (see S1 Fig).

Among the 17 randomly selected *Leptospira*-positive wild animal samples, six rat samples were successfully amplified on five loci only (excluding *icdA*, which PCR failed) and five rat samples were successfully amplified on all six loci; they were all identified as *L*. *interrogans* CC02 or ST02. Of note, we failed to amplify DNA at *icdA*, *lipL32* and *lipL41* loci for the rat sample that showed an alternative *secY* allele.

Among the 22 *Leptospira*-positive domestic animals, none were successfully amplified on all six MLST loci. However, produced sequences revealed a perfect match between *L*. *interrogans* genotyped from two dogs and from clinical samples. As for the first dog, the six-loci concatenated sequence showed close identity with ST34 but a 14 nucleotides long sequence was missing on *LipL41* locus, while for the second dog, the sequencing of four loci revealed relatedness to ST02 (four alleles in common; *adk* and *LipL41* non sequenced, see S2 Table).

As highlighted above, the *L*. *borgpetersenii*-infected samples were hardly detectable by PCR and could not be fully genotyped, likely because of low bacterial loads; thus the phylogenetic tree based on the concatenated sequences of the MLST loci included *L*. *interrogans* samples only (S1 Fig).

## Discussion

Although the mean prevalence of renal carriage among rats on Reunion Island (36.3%) was close to that reported in *R*. *rattus* on Mayotte (29.8%) [11], the genetic data reported in the present study reveal a striking contrast between the two islands: the rich *Leptospira* genetic diversity (at least 4 species with rather balanced representation) reported in rats from Mayotte, also found in human cases, contrasts with the low *Leptospira* genetic diversity reported herein in humans, rats and shrews on Reunion Island: though we identified three *Leptospira* species, *L*. *interrogans* represents 96.6% of clinical samples, 95.8% of positive animal samples and 100% of rat samples.

Other studies have reported a low genetic diversity among locally circulating *Leptospira* infecting local rat populations [25,51–53]. In contrast to earlier studies in New Caledonia [54], New Zealand [55] or Argentina [56], showing the carriage of *L*. *interrogans* or *L*. *borgpetersenii* in black rats (*R*. *rattus*), and the absence of *L*. *borgpetersenii* in Norway rats (*R*. *norvegicus*), *L*. *borgpetersenii* was absent in rats or shrews in our large sample of wild small mammals but was identified in a single mouse. The role of mice as maintenance hosts for *L*. *borgpetersenii* serogroup Ballum is largely recognized [1,3]. The perfect identity between *lipL32* fragments from a single mouse and one of the two clinical samples positive for *L*. *borgpetersenii* highlights mice as a possible reservoir of pathogenic *Leptospira* at risk for humans on Reunion Island. However, this result should be interpreted cautiously as a single locus might not be resolutive enough as to infer *Leptospira* species; indeed, *LipL32* sequences of *Leptospira borgpetersenii* and *Leptospira weilii* have been shown to be indistinguishable [57]. Further investigations targeting other loci with higher nucleotide polymorphism are needed to ascertain this point.

A different *L*. *borgpetersenii* infecting the second clinical case suggested the existence of a second potential reservoir host. A bat species endemic to Reunion Island, *Mormopterus francoismoutoui*, has been demonstrated as a *L*. *borgpetersenii* carrier in urine [50]. However, the comparison of *rrs2* sequences from our two clinical samples infected with *L*. *borgpetersenii* to GenBank sequences obtained from this endemic bat species (n = 12) showed a low degree of genetic relatedness (Fig 3), thus excluding bats as a source of contamination leading to overt clinical leptospirosis in humans. A serological survey conducted in 2009 showed that up to 32% of cattle were seropositive for leptospirosis in Reunion Island, with Sejroe reported as the main circulating serogroup [30]. Our investigation finally identified cattle as a potential reservoir of *L*. *borgpetersenii* at risk for humans, as one *L*. *borgpetersenii* infecting a cow was found related to the *L*. *borgpetersenii* infecting a clinical case (perfect identity between 434 bp *lipL32* fragments). As for mice, further investigation is needed in order to verify a potential transmission between cattle and human, possibly through an environmental maintenance of pathogenic leptospires [58].

Apart from the two *L*. *borgpetersenii* alleles common to human cases of leptospirosis and one cow or one mouse, at least two additional *L*. *borgpetersenii* haplotypes were identified in cows only, indicating a higher *Leptospira* diversity in cattle than in any other animal investigated on the island, which was already evidenced in other settings [59]. Our experience and previous published work [60,61] have highlighted recurrent failure to detect and amplify specifically *L*. *borgpetersenii* strains. Consequently, acute clinical cases related to this species might be underestimated.

The *L*. *kirschneri* found in two mice was not identified in any of our clinical samples on Reunion Island, whereas this species has been reported in clinical infection in Mayotte [11,12] as well as in other countries [2]. The reasons that may account for this difference are unknown. As mild infections not requiring hospitalisation or spontaneously healing cases are probably being underdiagnosed and underreported, it would be worth exploring whether these cases are associated with infection with other *Leptospira* species.

At an infraspecific level, we identified two clusters of *L*. *interrogans*, referring to sequence types ST02 and ST34 (http://www.pubmlst.org/leptospira/). International isolates previously published on the pubmlst.org database showed ST02 to be isolated from Belgium and Brazil, while ST34 was isolated from Jamaica and India. The second (minor) cluster, ST34, identified in six clinical samples, could not be detected in any of the five wild small mammal species targeted by our study. Additional sampling of domestic animals interestingly highlighted stray dogs as possible carriers of both *L*. *interrogans* STs found in human acute cases. Dogs have been repeatedly pinpointed as involved directly or indirectly in human epidemiology (contamination from dogs to humans, or from a common environmental source) [62–66]. For instance, a serosurvey conducted on Reunion Island in 2009 identified Canicola and Icterohaemorrhagiae as the major serogroups (43.5% and 21.7% respectively) in seropositive stray dogs, while Sejroe, Panama, Tarassovi and Ballum serogroups were also identified [29]. On the other hand, up to 17% of clinical cases were found to be infected with serogroup Canicola [28], suggesting that dogs, as the reservoir for this serogroup, are the source of contamination on Reunion Island. The importance of dogs in the epidemiology of the disease, if confirmed on Reunion Island, might be related to the lack of mammals diversity locally, as suggested on the island of Barbados [67].

An epidemiological survey conducted by CIRE-OI (Regional Office of French Institute for Public Health Surveillance in Indian Ocean, see [13]) indicates that, regarding the six patients infected with *L*. *interrogans* ST34, (i) five were actually living in the eastern, windward humid coast of the island and (ii) for those five cases, the disease was associated to leisure activities in the eastern side rivers and waterfalls. Noteworthy, of 56 *R*. *rattus* positive for *Leptospira* trapped in sites near rivers in the eastern transect (Fig 1), none of the 53 available sequences corresponded to *L*. *interrogans* ST34. Hence, it seems very unlikely that *R*. *rattus* acts as the reservoir of *L*. *interrogans* ST34.

In the end and as expected, our analyses stress black and Norway rats as reservoirs of *Leptospira* lineages of medical importance on the island. However, we were able to identify other animal species likely playing a role in the local epidemiology of human leptospirosis: mice, cattle and stray dogs. Noteworthy, the very low infra-specific diversity of *L*. *interrogans* infecting our infected samples did not enable us to assess the respective contribution of rats versus dogs to acute human infection with *L*. *interrogans*. *Leptospira* isolation from animals and humans would help to better assess the epidemiological links between humans and animals. Our conclusions are weakened by PCR and/or sequencing failures, likely resulting from different primer annealing efficiencies depending on the *Leptospira* species used as template (supported by our incomplete results on cattle and mice samples infected with *L*. *borgpetersenii*), or from low renal carriage, as suggested by high *Ct* values (*e*.*g*. *Ct* >30 in rats or >37 in dogs with undetermined infecting *Leptospira*, see S3 Table). Although time-consuming and fastidious, *Leptospira* culture needs to be carried out systematically, whenever possible, as previously suggested by other authors [68].

The high infection prevalence of cows with *L*. *borgpetersenii* raises another issue: the available human vaccine against leptospirosis currently proposed on the island target only *L*. *interrogans* species serogroup Icterohaemorrhagiae, while Sejroe was reported to be the main serogroup circulating in cattle in Reunion Island in 2009 [29]. This means that the vaccination of the ranchers’ population, at risk for leptospirosis for being in close contact with cows, might be inefficient against any other serogroup likely carried by livestock, in particular the one associated with *L*. *borgpetersenii*.

Pigs are another potential reservoir that deserves attention. Leptospirosis is a common disease of swine throughout the world and can be a significant cause of reproductive loss [69]. None of the 22 pig samples included in the study tested positive to *Leptospira*, while a serological survey conducted in 2009 on Reunion Island showed that up to 47.2% of pigs were seropositive for leptospirosis [29]. These two observations are not contradictory. First, the acute infection of an animal with leptospires does not prejudge its ability to develop a bacterial biofilm in the renal tubules and to shed the bacteria into the environment. Hence, serological data and PCR data on kidneys may be discrepant. Second, the kidney samples used in our study were collected from growing-finishing pigs under one year old, a potential age bias that might have affected our findings. Indeed, a proper assessment of pigs as reservoirs of leptospires at risk for human health requires the screening of older animals sampled from different farms as well as from family breedings throughout the entire island.

It is hypothesized that *Leptospira* were introduced onto islands with their animal hosts, and that a variable number of introduced strains have adapted to the new local environment and available hosts [14]. On Reunion Island, the fact that *L*. *interrogans* is almost clonal in rats is in favour of a recent leptospiral introduction on the island concomitant with the introduction of rats, back to the 16^th^ century. Leptospires detected in dogs, mice and cows show higher genetic diversity, which could result from multiple introduction events of infected animals of different species (for cows, there has been multiple importations from France of both Holstein Frisonne and Montbéliarde until 2008) or from distinct geographical origin, carrying different *Leptospira* lineages. We suggest that in contrast to Mayotte where the high diversity of *Leptospira* in humans and animal reservoirs is most likely related to (older) introduction events of infected hosts, potentially from countries with high host endemicity associated with *Leptospira* diversity [26], on Reunion Island, the narrow diversity of *Leptospira* might reflect more recent and/or fewer host introduction events. This is partly supported by the fact that fifteen of the seventeen STs from Mayotte strains have not been previously identified [12], contrary to STs from Reunion Island clinical cases that have already been identified worldwide. Previous studies have suggested that *Leptospira* from Mayotte and Madagascar are genetically closely related, and that *Leptospira* identified in Mayotte are probably mainly derived from *Leptospira* from Madagascar, possibly introduced in Mayotte via their hosts [14]. Although no genetic information is available so far on pathogenic *Leptospira* prevalent on Comoros, the serological profiles in humans from Comoros are comparable to those depicted in Mayotte, and noteworthy, antibodies from the serogroup Icterohaemorrhagiae are not detectable [20]. These findings contrast with those from human leptospirosis in Reunion Island and the Seychelles, where the Icterohaemorrhagiae serogroup is most common [70]. Hence there is compelling evidence that leptospirosis epidemiology might be similar along the Comoros, Mayotte and Madagascar axis and associated to indigenous or endemic *Leptospira* lineages, but different from that prevalent on Reunion Island and potentially Seychelles and Mauritius, where Icterohaemorrhagiae serogroup is dominant in clinical cases [15,16,21]. The near clonality reported herein in rat-borne *Leptospira* on Reunion Island is highly suggestive of recent pathogen introduction. This has to be addressed in the future by similar molecular analyses of *Leptospira* isolates from the other SWIO islands for which almost no molecular data are available so far, i.e. Mauritius and Seychelles.

## Supporting Information

S1 Table GenBank accession numbers of the nucleotide sequences used in this study. (XLSX)

S2 Table List of samples investigated with successfully amplified MLST loci and non-successfully amplified (NA). For each sample, the table provide information on the host species, the infecting *Leptospira* species, the allele identifiers (numbers assigned to unique sequences) for each locus and the corresponding allelic profile or sequence type (ST) when available. * Nearest match for the sequence type considering the available loci.
(XLSX)

S3 Table List of *Leptospira*-positive animal samples. For each sample, the table provide Ct values obtained by real-time PCR diagnosis, the sample site, the host species and the infecting *Leptospira* species. *RR*: *Rattus rattus*; *RN*: *Rattus norvegicus*; *SM*: *Suncus murinus*; *MM*: *Mus musculus*; *TE*: *Tenrec ecaudatus*.
(XLS)

S1 Fig Phylogeny of Reunion Island samples and reference strains using 5 MLST loci. *Leptospira interrogans* phylogeny based on a concatenated 2292 bp sequence. Black legends indicate reference strains, red legends indicate samples from Reunion Island, blue legends indicate a clinical sample from Mayotte. GenBank accession numbers are provided in S2 Table. CC: clonal complex (sequence type [ST] and their single locus variants).
(TIF)
