# Supplementary figures and images for: Human Leptospirosis on Reunion Island, Indian Ocean: Are Rodents the (Only) Ones to Blame?
Source: PLoS Negl Trop Dis. 2016 Jun 13;10(6):e0004733. doi: 10.1371/journal.pntd.0004733 (PMC4905629; doi:10.1371/journal.pntd.0004733)

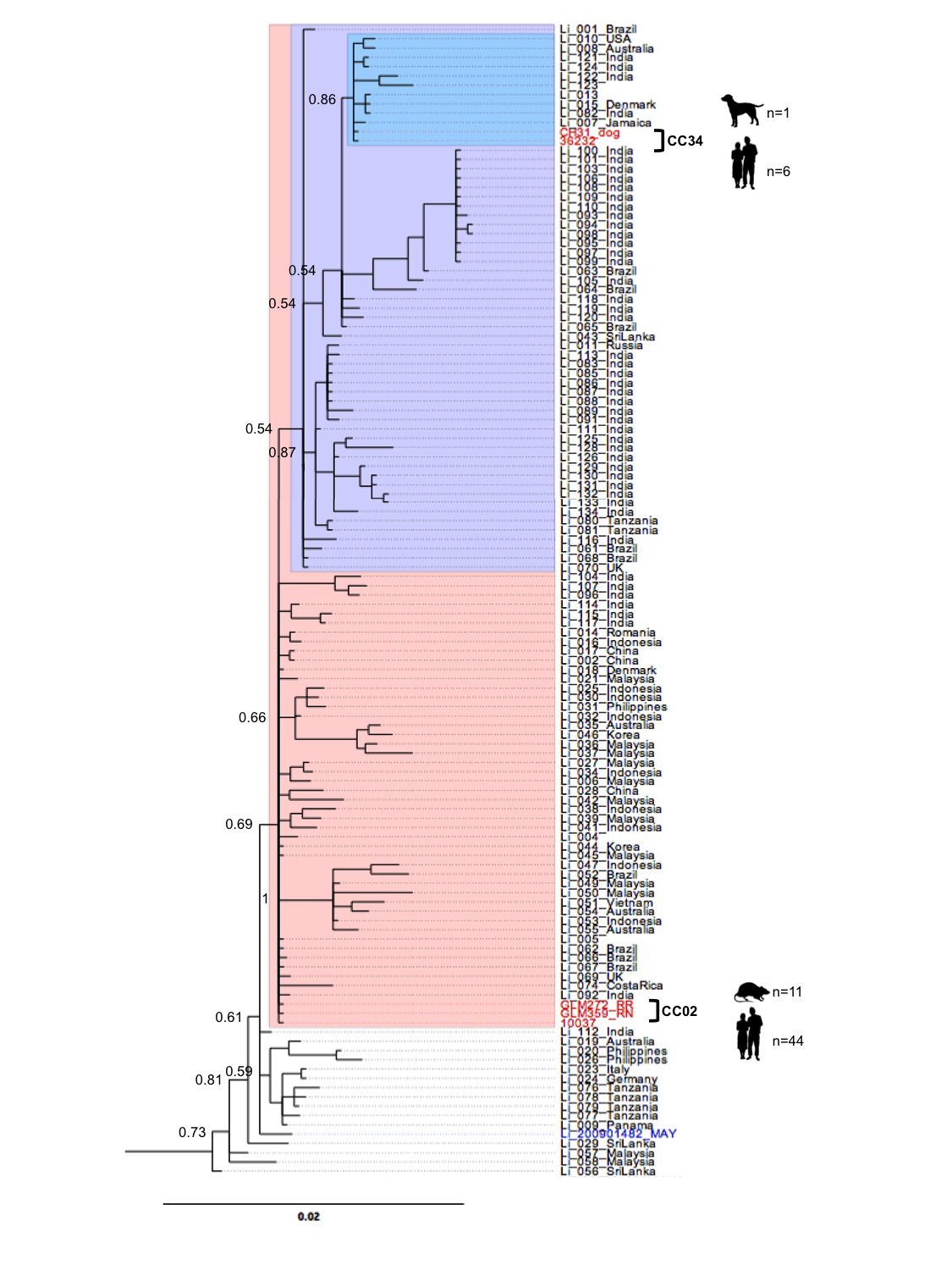

Supplement: S1 Fig — Black legends indicate reference strains, red legends indicate samples from Reunion Island, blue legends indicate a clinical sample from Mayotte. GenBank accession numbers are provided in S2 Table. CC: clonal complex (sequence type [ST] and their single locus variants). (TIF) [file pntd.0004733.s004.tif]
